# An Account of the North Kensington Friendly Workers among the Poor

**Published:** 1892-09-10

**Authors:** C. Furley-Smith


### THE CURE OF POVERTY,

### AN ACCOUNT OF THE NORTH KENSINGTON FRIENDLY WORKERS AMONG THE POOR

—(*continued from page* 384).

By Mrs. C. Furley Smith.

The first thing to be done was to get a reliable list of all the poor in the district. This was supplied by the district visitors of the various congregations, but supplemented by the visits paid by the Secretary and the friendly workers who, apart from the existing agencies, put themselves at the command of the Association. These were supplied with books containing the following questions to be answered:—

General Remarks.

Name of Street or Court and number of House:
 Nature of Tenement:
 Condition of Families Resident:
 Cases of Sickness, Doctor or Institutions in Attendance:

Particular Inquiries.

Name of Street or Court and number of House:
Nature of tenement:
Name of occupier:
Number, age, and sex of inhabitants:
 Place of worship:
 Visitor, if any.
Number of beds:
Wages of each of the inhabitants:
 Benefit societies or insurance?
Nature and place of employment of the inhabitants:
 If children, where at school.
 Previous residences.
Length of occupation of tenement by present occupier:
Rent:
 In arrear or not:
Name and address of landlord or deputy, or both:
Nature of repairs, if any, within the remembrance of the present occupier:
Complaints, if any, and to whom made, and with what result:
 Nature of special help wanted?

When these books were filled up they contained a fairly complete history of every family dealt with. When any of these applied for help a second time it was easy to refer to this record to find out their history and what assistance they had had before. The great aim was to supply work. There are slack times in so many trades that in a poor locality you will in the winter find always a number of men who are anxious for odd work of any description, and a very large number of women who, from one cause or another, must take upon themselves the charge of providing for their families. A list was formed of these; and besides the efforts made by the Secretary to find work for each case, the inhabitants of the district who were in want of such occasional assistance were invited to apply to the office at 28, Colville Square. A large number of women were thus provided for. Women are more adapted to casual work than men; they are accustomed to all manner of household employments, and can thus, in the common phrase, turn their hand to anything. The majority found employment as charwomen, others as seamstresses; those who had been in good domestic service before their marriage were able to do an occasional day's cooking when required, or to take the place of servants who had fallen ill or left their situations suddenly. Speaking generally, the women who asked for help were not nearly so difficult to deal with as the men.

A man, as a rule, knows his trade and knows nothing more. If he cannot get work in it he is at a standstill. But the most difficult subject to "place" anywhere was found to be the old soldier, who, when his time expires, is sent off with a pension on which only the wildest dreams of frugality would imagine he can live. He knows no trade, and he is too old to learn one. He may get employment as a commissionaire, but failing this, his outlook is very gloomy. The recent ordinance of the Postmaster-General, by which a number of post office appointments are available for time-expired soldiers, will help to solve the difficulties of what is undeniably a national responsibility, and even before this regulation was promulgated Mr. Mackenzie was frequently in communication with the postal authorities regarding the employment by them of some of the old soldiers living in North Kensington. In the case of elderly couples the post of caretaker of an empty house was often obtained, and occasionally employment was found for a family in the country. Many workers among the poor complain that London people will not go into the country, even though there they would have manifestly better conditions of life and labour. How paralysing such obstinacy is need not be detailed. The philanthropist often stands helpless between the overcrowded towns and the depopulated country, vainly beseeching the surplus of the one to fill the emptiness of the other, and baffled either by the craving for the variety and excitement that are found in even the poorest city life, or by that poverty of physique which makes townspeople so helpless out of their own narrow round. In face of this complaint it is satisfactory to know that in North Kensington no such difficulty was found in the cases where work was found outside the limits of London.

It must not be supposed, however, that the office in Colville Square was simply a Labour Bureau of casual workers, open to any who chose to apply. There are in every district a number of poor who—may one say it frankly?—have no right to be relieved by other than the parochial authorities. In cases where poverty is the direct and obvious result of their own improvidence, self-indulgence, and sin the Union is the only place. The North Kensington Association of Friendly Workers had to do more with the hopeful poor—those whose condition was the result of special circumstances, and whom the help thus given might be expected to render self-supporting. It was necessary that money should not be expended on those who would simply consume it and ask for more, nor on those varieties of the genus tramp who, whenever they hear of help being given in a particular locality, come and squat there on the chance of getting some of it. To circumvent these gentry the following rules were laid down:—

1. The Committee will meet from time to time as occasion requires.

2. No applications for assistance of any kind will be received except through a responsible society, or individual, or known householder. The necessary forms for cases not already on register, will be supplied on application to the Honorary Secretary.

3. Before a grant is made to a charitable institution or agency, the recognised representative of such institution or agency must furnish the Committee with full particulars of each individual case, and also undertake to apply the grant in accordance with these rules and regulations.

4. A register will be kept (for the use of the Committee) of the names and addresses of all persons on behalf of whom application is made, with the names of the friendly workers recommending them, and with particulars of the assistance given.

5. Assistance from this fund will be limited to persons who have resided in the above specified district of North Kensington for not less than 12 months prior to the date of application, with the exception of very urgent cases.

6. No relief in money will be given unless it be shown to the satisfaction of the Committee that employment cannot be obtained.

7. Relief in money will only be given in the following cases:—

(a). When the distress is shown to be exceptional.

(b). When it is required to keep a man on his benefit club, or reinstate him where his membership has recently run out.

(c). When it is required to redeem necessary articles that have been pawned, or to provide tools.

8. When relief in money is granted the friendly worker on whose recommendation the grant was made, or the visitor, or member of the Committee who acts as Almoner, will be required to see, as far as may be possible, that it is properly applied, and that the case is visited again within a week from payment.

9. If a specially urgent case be reported to the Secretary, and the urgency is confirmed by letter of a visitor, or member of the Committee who has personally visited the case, such relief as is consistent with these rules may be supplied forthwith, the same being reported to the next meeting of the Committee of Friendly Workers.

The details the Committee required for their guidance were contained in the answers to the following table of questions, but in a vast number of cases the Secretary was, from his personal knowledge of the applicants, already cognisant of many of the particulars, or could ascertain them by reference to the census books of the Society.

Name....................................................................
Address................................................................
How long Resident...............................................
Occupation or Trade ............................................
Age ........................................................................
Weekly Wages when in Work .............................
How long out of Work .........................................
Wife's Age, Occupation, and Earnings ...............
Number in Family ..................................................
Ages of Children....................................................
Where at School......................................................
Children in Employment, and Wages ..................
How long a Resident in North Kensington ..........
Character of Assistance recommended ...............
Amount of Relief from Guardians .......................
Amount of Relief from Local Charities ...............
Whether a Member of any Benefit Club, or Insurance Society, and arrears of Subscription ..........................
Amount of weekly rent .........................................
Is any Rent owing, and if so, how much .............
Are any articles or Tools in pawn .......................
Is there any prospect of employment, if so, when and where...................................................................
Name and Address of last Employer ....................
Visitors, if any ......................................................
Clergy Reference ..................................................

In all cases of a man being out of employment it was necessary to find out the reason of his idleness. To this end the man's explanation was heard, and a statement of it sent to the late employer for confirmation. It is satisfactory to know that in most cases the statements of master and man coincided. While a man who had lost his situation through discourtesy and ill-temper, or, perhaps, a burst of drunkenness, could not expect to be received on the same terms as one who had suffered merely from slackness of work, those who had some faults of their own to thank for their misfortunes, were not always discarded. A conscientious attempt was made to relieve and elevate them by means of whatever good qualities they possessed, and the results were, as a rule, most satisfactory. But there is one form of deception that is frequently practised on every charitable organisation. A man has been out of work for some time, and in the poverty resulting he has been driven to pawn his tools. He comes to say that if only he could get his tools out Mr. So-and-so would give him work. Sometimes this is a true story, sometimes it is false. To find out when the statement was true, the North Kensington Association had a form printed, which was sent to the employer whose name had been mentioned. It ran as follows:—

To Mr.________________________

Dear Sir,

The undermentioned states that you can give him Employment at once.________________________

________________________________________

Kindly inform me below if this is correct.

Yours faithfully,

DONALD MACKENZIE,

*Honorary Secretary and Agent.*

---

Name________________________________

Address______________________________

REPLY—

Similar forms were used for sending to any clergyman or other person whom an applicant had named as a reference, and for inquiring of the managers of church and other charitable funds if any help were being given from them.

By these means the *bonâ fides* of applicants was tested, and the duplication of charities prevented. In cases where a church or some other body was giving help the Association occasionally supplemented it; but always with the express intention of speedily putting the recipients in a position above the need of help. To this end it is often necessary that the alms should be comparatively large. The dole that just serves to keep body and soul together very rarely elevates those who receive it; they take and spend it, and are in as great need as ever. Half-a-crown a week, given for a year, will hardly keep a man from starving, while half the total sum, given at once, may enable him to get tools, keep a home together, set up in business in some small way, move to some locality where work is more plentiful, or in some other way raise him above the need of charity.

(*To be continued.*)

---

We cannot wonder that in America the cry against the enormous increase in the numbers of emigrants flocking to their shores has arisen. What was once not only a benefit, but an actual necessity, is now degenerating into an actual curse. Skilled mechanics and labourers, and the hardy and competent of all sorts, sought for a wider field for their energies in the earlier years when the tide flowed westward; now, to quote an American paper, "the influx is becoming intolerable and alarming." Those who now arrive daily from Europe are described as "a burden or a pest in their own countries; they are dumped upon American shores, and cling to the cities as offensive fungi or spread throughout the country as weeds, to the injury of wholesome growths." Those who watch the sequence of events cannot fail to see that America will not for ever consent to act as the "dust-heap of Europe," but the day when she closes her doors will be a bad one for the nations, many of whose sons have learnt to look upon her as their last hope.

